# Exploring the Limits of Biological Complexity Amenable to Studies by Incoherent Neutron Spectroscopy

**DOI:** 10.3390/life12081219

**Published:** 2022-08-11

**Authors:** Eugene Mamontov

**Affiliations:** Oak Ridge National Laboratory, Second Target Station Project, Oak Ridge, TN 37831, USA; mamontove@ornl.gov

**Keywords:** biosystems, molecular-level dynamics, incoherent neutron spectroscopy

## Abstract

The wavelengths of neutrons available at neutron scattering facilities are comparable with intra- and inter-molecular distances, while their energies are comparable with molecular vibrational energies, making such neutrons highly suitable for studies of molecular-level dynamics. The unmistakable trend in neutron spectroscopy has been towards measurements of systems of greater complexity. Several decades of studies of dynamics using neutron scattering have witnessed a progression from measurements of solids to liquids to protein complexes and biomembranes, which may exhibit properties characteristic of both solids and liquids. Over the last two decades, the frontier of complexity amenable to neutron spectroscopy studies has reached the level of cells. Considering this a baseline for neutron spectroscopy of systems of the utmost biological complexity, we briefly review what has been learned to date from neutron scattering studies at the cellular level and then discuss in more detail the recent strides into neutron spectroscopy of tissues and whole multicellular organisms.

## 1. Introduction

Neutron spectroscopy occupies a special place among spectroscopic techniques. Some of the advantages of neutrons as a spectroscopic probe pertain equally to studies of organic and inorganic systems. For example, among the common particle probes, neutrons produced at neutron scattering facilities not only have the wavelengths comparable to typical intra- and intermolecular spacings, similar to photons at synchrotron sources, but also, unlike photons and electrons, they possess relatively low energies. The typical energies of such neutrons, in the meV to hundreds of meV range, are comparable with inter- and intra-molecular vibrational energies. Moreover, small energy exchanges with a sample related to the relaxational dynamics are easily measurable with such a low energy probe. State-of-the art inelastic x-ray scattering resolving ~10^−6^ changes in the energy of the ~keV photons can measure ~meV dynamics in a sample, whereas inelastic/quasielastic neutron scattering (INS/QENS) resolving merely ~10^−2^–10^−3^ changes in the energy of the ~meV neutrons can measure ~µeV dynamics in a sample. Alternatively, state-of-the-art neutron spin-echo techniques resolving ~10^−6^ changes in the energy of the ~meV neutrons can measure ~neV dynamics in a sample. Thus, neutron scattering can provide information simultaneously on the spatial (via the scattering momentum transfer, Q) and temporal (via the energy transfer, E) aspects of the dynamics in a sample, both vibrational and relaxational.

Particularly pertinent to organic samples, the scattering cross-section of neutrons, unlike that of photons and electrons, does not increase strongly and systematically with the atomic number, making neutron scattering relatively sensitive to light elements. The reason for this is the interaction of neutrons not with the electron shells, but the nuclei. This is critical for the stability of biological samples in a neutron beam (no radiation damage by neutrons, as opposed to photons and, especially, electrons) and the capability of probing relatively thick, biologically relevant samples. As a side benefit, bulky sample environment cells (e.g., for high pressure studies) could be readily penetrated by neutrons.

Finally, the fact that neutrons can be scattered by nuclei both coherently and incoherently, thus probing, respectively, collective and single-particle motions in a sample, uniquely benefits studies of biological systems. By far the largest among the elements, neutron scattering cross-section of protons is predominantly incoherent, whereas that of deuterons is much lower overall and comprised of the more comparable coherent and incoherent contributions. In structural studies measuring the scattering intensity as a function of Q (neutron diffraction and, especially, small-angle scattering), the opposite signs of the coherent scattering length for protons and deuterons are commonly relied upon for contrast matching, while the incoherent scattering merely gives rise to the Q-independent background. On the contrary, in the spectroscopic studies probing the scattering intensity as a function of both Q and E, the incoherent scattering from the protons in the sample tends to dominate the scattering signal, although the contribution from the coherent scattering may become more appreciable, e.g., at the Q values corresponding to the structural factor maxima. Therefore, the INS/QENS scattering signal, I(Q,E), measured from a native-state biological sample at ~0.1 Å^−1^ < Q < ~1.0 Å^−1^, will be predominantly due to the proton-weighted incoherent neutron scattering from all the molecules in the sample, representing the averaged single-particle proton dynamics. If a deuterated component is used (e.g., the H_2_O in the native-state sample is replaced with D_2_O), then the contribution from this component to the overall (predominantly incoherent) scattering signal becomes greatly reduced. However, depending on the spectral weights of the components’ scattering signals, this does not necessarily mean that the deuterated component becomes invisible. For example, if, in the native sample comprised of hydrated/solvated biomolecules, the scattering signal was completely dominated by the H_2_O, then, in the H_2_O-replaced sample, both the D_2_O and non-aqueous (protonated) components may provide comparable contributions to the scattering signal.

The use of D_2_O-based buffers is common in neutron scattering studies of model systems, such as aqueous solutions of proteins or lipids. However, with the increasing complexity, biological systems tend to show less tolerance of D_2_O. Additionally, the dynamics associated with collective fluctuations in water greatly differ from those originating from single-particle motions [1]. Short of elaborate neutron spectroscopy experiments based on polarization analysis, which holds great promise for distinguishing between the collective and single-particle molecular-level dynamics in aqueous and biological systems [1], the scattering signal from deuterated components represents different dynamics from those that would be exhibited by their protonated counterparts. Fortunately, despite a strong scattering signal from H_2_O-based buffers, their use under in vivo conditions can be possible because the spectrometer’s energy and scattering momentum transfer ranges can be chosen to filter out the strong contribution from the H_2_O dynamics [2].

Neutron spectroscopy of proteins [3] and lipid membranes [4] was pioneered more than 30 years ago and has been widely adopted since. A few of the many prominent concepts introduced or developed in relation to neutron scattering studies of biomolecular dynamics include temperature-dependent dynamical transition [3], protein “softness” [5], protein energy landscape [6,7], and the effects of water (or other solvents) on the dynamics of biomolecules [7,8,9]. There are many recent reviews, both focused [10] and broad in scope [11], that discuss the applications of neutron scattering, including inelastic scattering, in biological sciences, which are predominantly concerned with model systems that are oftentimes amenable to detailed analysis. On the contrary, in the current review, we would like to discuss the potential of neutron spectroscopy for studying real-life biological systems, from the cellular level and up. Although much less developed compared to measurements of model biosystems, inelastic neutron scattering from real-life biological samples holds promise for a better understanding of biological complexity. We do not intend to concentrate on the details of the incoherent neutron spectroscopy methods, as they have been discussed in several books and some very recent review articles [12]. Instead, we focus on interpretation of the information provided by neutron spectroscopic measurements of complex biological systems.

## 2. Incoherent Neutron Spectroscopy: Cellular Level

Neutron spectroscopy of cells was pioneered almost two decades ago [2]. Early studies largely concentrated on the dynamics of intracellular water, e.g., in *E. coli* [13], red blood cells [14], various bacteria including extreme halophiles [15,16,17], and cyanobacteria [18]. A recurrent topic of these investigations has been the distinction between cytoplasmic water, with the dynamic properties resembling those of bulk water, and much slower hydration water, in direct contact with the cellular constituents. The detailed picture can be more nuanced: e.g., only the diffusion coefficient of the cytoplasmic water molecules, but not the residence time between their successive diffusion jumps, can be close to the bulk water value, or the hydration water could be subdivided into various populations with different dynamics. Nevertheless, the distinction between the “free” cytoplasmic and “bound” hydration water is generally accepted. Measurements of intracellular water dynamics are aided by its strong, usually dominant, incoherent scattering signal.

In parallel to studies of intracellular water, measurements of intracellular biomacromolecular dynamics have been reported. Many such measurements have been performed on the systems previously (on concurrently) employed in intracellular water dynamic studies, e.g., in *E. coli* [19], red blood cells [20], various extremophiles [21,22,23,24,25], and cyanobacteria [26,27]. Various degrees of suppression in the dynamics of the intracellular components have been observed. For example, measurements of GroEL protein overexpressed in living deuterated *E. coli* cells revealed a four-fold and a two-fold decrease in the protein’s global diffusivity and internal dynamics, respectively, compared to the same protein in a buffer aqueous solution [28]. A study of the effect of crowding in *Thermococcales* cells revealed that, in contrast to the impact of crowding on pure proteins in solution, the release of crowding constraints on proteins in the cells leads to an increase in the rigidity and a decrease in the high-pressure sensitivity [29]. This example again clearly demonstrates a non-trivial role of the cellular environments that cannot always be replicated in simplified model systems. Neutron spectroscopy measurements of dynamics in bacterial spores have also been reported [30].

Recently reviewed [12,31] neutron spectroscopy studies of cancer cells represent a distinct line of research. In a pioneering study [32], it was reported that exposure of human metastatic breast cancer cells to an anticancer drug, cisplatin, induced distinct responses in the non-hydrating cytoplasmic and hydrating water molecules, which exhibited, respectively, suppressed and enhanced diffusion dynamics. A subsequent study of the effect of cisplatin and spermine on human osteosarcoma cells revealed the same trend, of the drug-induced lower mobility in the bulk-like water and higher mobility in the confined water [33]. The latter effect was explained on the grounds that a conformational rearrangement upon drug binding leads to a disruption of the previously highly structured hydration shell. The impact of anticancer drugs on the extracted DNA was investigated in separate QENS experiments [34,35]. A more recent study by the same group involved a comparison of normal and cancerous human cells; the most remarkable observation made was that, in the cancer cells, a higher fraction of water molecules contributed to the dynamics detectable by QENS [36]. A combined INS/QENS study of a breast cancer cell line before and after treatment with the drug paclitaxel that primarily interacts not with the DNA, but the cellular membrane, revealed a drug-induced increased population of the confined water [37]. Since the vibrational modes specific to protein and DNA components were not altered by the drug, the results indicated that changes in the dynamics and structural organization of the water molecules could be observed before any detectable changes in the other cellular components became evident.

**Figure 1:**
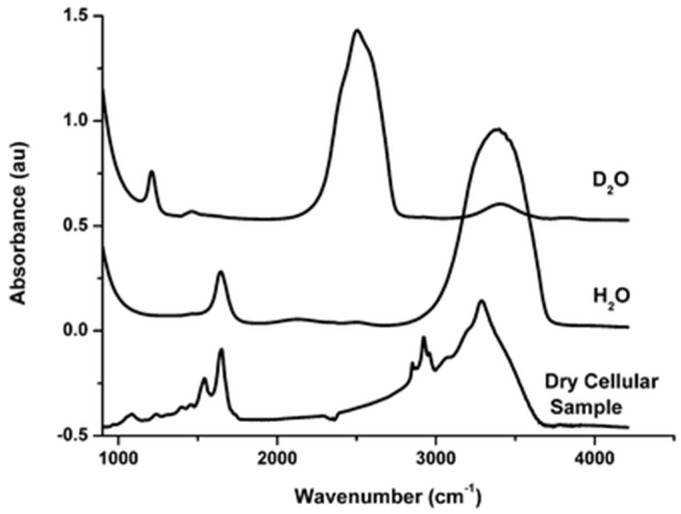
FTIR absorption spectra of H_2_O and D_2_O compared with a typical cell spectrum. The spectra have been rescaled and an offset introduced for clarity. Reprinted with permission from [39]. Copyright 2011 The Royal Society of Chemistry.

Neutron spectroscopy experiments at the cellular level have become more common lately, yet often remain challenging from the standpoint of the scattering signal analysis. They can sometimes make use of D_2_O to reduce scattering from the deuterated components. Analysis of the Q-dependence of the scattering signal is essential in distinguishing between the dynamics of cytoplasmic water, hydration water, and other cellular constituents. The latter are often assumed to be associated predominantly with the cellular proteome. Indeed, biochemical compositions reported for systems such as *E. coli* bacteria and mammalian cells suggest [38] that proteins, DNA/RNA molecules, and lipids/lipopolysaccharides are all present in sufficient quantities, with the proteins accounting for the largest fraction by weight (of the dry material). Analytical techniques traditionally associated with single-cell spectroscopy, such as Fourier-Transform Infrared (FTIR) spectroscopy, demonstrate that, besides the water modes, intra-molecular cellular spectra (an example is given in Figure 1) are largely composed of stretching vibrations of CH_2_ and CH_3_ groups, mainly contained in acyl chains of lipids, in the 3050–2800 cm^−1^ spectral region and vibrations of peptide bonds (C=O and C–N stretching, N–H bending modes) at ∼1650 cm^−1^ and ∼1540 cm^−1^ (named Amide I and Amide II, respectively) [39,40]. For the energy scale conversion, 1 meV is ca. 8 cm^−1^.

While the relative intensities of various vibrational modes would differ between IR and inelastic neutron scattering measurements, scattering signals from the water, proteins, and lipids are all expected to contribute to INS spectra measured from cellular cultures. In QENS experiments that measure scattering intensities near the elastic line (energy transfer ~ 0), a sample component’s contribution to the scattering signal is proportional to the component’s mole fraction and the component’s total scattering cross-section (dominated by hydrogen atoms). Therefore, the contributions to QENS signal from typical cells are expected to be in the order of water > proteins > lipids, unless partial or full substitution with D_2_O has been performed. Specialized cells, such as adipocytes, may exhibit a different order of spectral weights of the cellular components.

## 3. Incoherent Neutron Spectroscopy: Tissues

INS measurements on an array of samples, including plants and animal tissues, were reported almost 20 years ago [41]. The scattering signal was dominated by bulk-like water, intracellular, and, possibly, extracellular. Nevertheless, considering distinct spectral signatures of the interfacial water, it was possible to quantify its content, which showed significant variations among the samples of various origins. Enabled by the penetrative power of a neutron probe, the measurements used tissue slices as thick as 3 mm. As is customary in INS, the measurements were performed at deep cryogenic (near liquid helium) temperatures, to limit temperature-dependent suppression of the vibrational signal. It took another decade until QENS measurements were used to probe room-temperature mobility of water in 0.05 mm thick slices of bovine neural tissue [42,43]. Again, two dynamic components, associated with “free” and restricted water populations, were reported. Neutron spectroscopy measurements of time- and temperature-dependent dynamics in germinating seeds were reported shortly thereafter [44].

**Figure 2:**
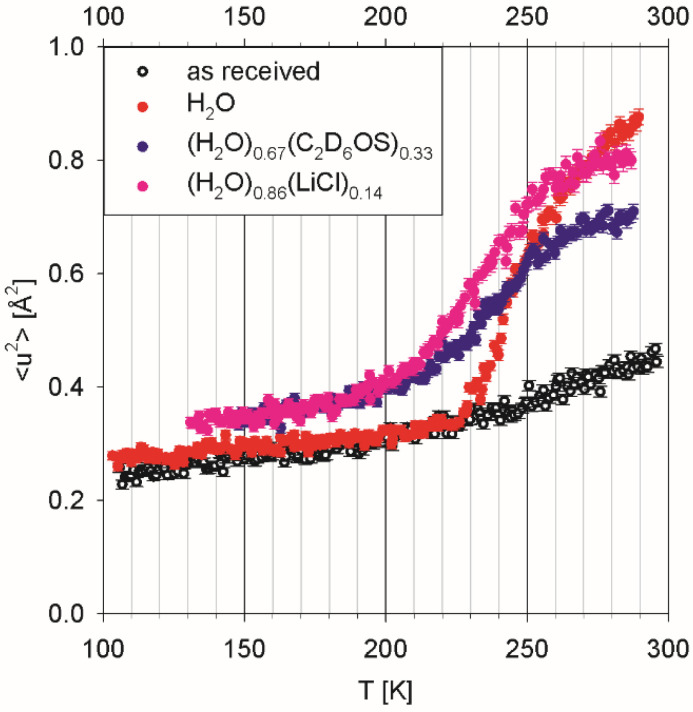
Mean-squared atomic displacement of hydrogen atoms in brine shrimp eggs, dry (as received) and hydrated with protonated aqueous solvents, as measured by energy-resolved “elastic” incoherent neutron scattering. Reprinted with permission from [46]. Copyright 2017 Elsevier.

QENS experiments on brine shrimp eggs [45,46] could be considered measurements of multicellular organisms, but not in a metabolically active state. For this reason, they may have more in common with tissue measurements. Females of brine shrimp (genus *Artemia*) may produce developmentally arrested embryos at the gastrula stage that are enclosed in a protective shell, known as cysts. The encysted embryos enter diapause, which is a state of greatly reduced metabolism. Anhydrous (dried) encysted brine shrimp eggs are of significant importance in commercial aquaculture. As illustrated in Figure 2, the eggs as received from a commercial supplier were not completely devoid of water, as evidenced by a small upturn in the temperature-dependent mean squared displacement plot above ca. 225 K. Indeed, they exhibited weight loss of ca. 8 wt. % in a test when placed in a 10^−3^ mbar vacuum at ambient temperature for 24 h.

Three separate batches of the eggs were hydrated using (a) deionized water, (b) an aqueous solution of deuterated dimethyl sulfoxide (DMSO) of eutectic composition, (H_2_O)_0.67_(C_2_D_6_OS)_0.33_, and (c) an aqueous solution of lithium chloride, (H_2_O)_0.86_(LiCl)_0.14_. Dimethyl sulfoxide is a commonly used cryoprotector employed to prevent freezing damage to living systems [47]. Aqueous solutions of lithium chloride are remarkably close to pure water in glass-forming properties and may enable low-temperature measurements of biomolecular solutions without resorting to nanoscopic confinement for freezing prevention [48]. The H_2_O-hydrated eggs demonstrated progressive water freezing, manifested in the intensity drop on cooling down, which gets completed at ca. 230 K. Continuous water freezing, as observed for this sample, is indicative of a distribution of the confinement pore sizes over the range of 2 to 10 nm. There is no evidence of intra-cellular cytoplasmic water in the hydrated eggs, which would exhibit freezing not far below 273 K. Adding a co-solvent (DMSO or LiCl) suppresses water freezing in the hydrated eggs, suggesting that co-solvent’s molecules penetrate the same voids as water molecules penetrate.

Complex samples such as tissues are typically measured in the native (H_2_O-hydrated state), which often makes the water scattering signal dominant. As illustrated by the latter example, different liquid probes can be used in the systems where cytoplasmic water is not present. In such cases, incoherent neutron spectroscopy studies may be conceptually similar to studies of fluids in confinement.

## 4. Incoherent Neutron Spectroscopy: Multicellular Organisms

The penetrative power of neutrons already plays a role in tissue measurements, because tissues can be challenging to prepare as sufficiently thin samples. The challenge associated with sample geometry would be exacerbated in measurements of living multicellular organisms, which could not be dissected. It is for this reason that living planarian flatworms placed in a 0.5 mm thick container were selected as potentially suitable candidates for a QENS experiment. Planarians are three germ-layer acoelomates with a solid body and no body cavity that also lack circulatory and respiratory systems. Emptiness of their digestive system is readily achieved by withdrawing food, without which they can survive for weeks. The inability of flatworms to survive even for a few hours in heavy water [49] required the use of H_2_O for their environment medium. Unlike with a photon-based, let alone an electron-based, probe, neutrons readily penetrate through a 0.5 mm thick sample composed of living planarian bodies and the water buffer to measure bulk, as opposed to surface, sample properties. Nevertheless, the effects due to the multiple scattering of neutrons in a hydrogenous sample of such thickness cannot be disregarded. Fortunately, addition of a constant term to the law describing the Q-dependence of the QENS signal broadening, first introduced to analyze the measurements of thick water samples [50], could be also successfully employed in analysis of QENS data collected from living planarian flatworms in H_2_O loaded in a 0.5 mm thick sample holder [51].

Approximately 30 flatworm specimens, each of a few millimeters in length, were gently placed into a cuvette-type aluminum sample holder with a plate insert utilized to make the inner holder 30 mm wide, 70 mm deep, and 0.5 mm thick, filled with ca. 0.6 g of deionized H_2_O. The resulting total mass of planarian specimens in H_2_O was ca. 0.8 g. The sample holder was not filled up to the top and not sealed to ensure continuous exposure of the sample to atmospheric air to prevent oxygen deprivation to the flatworms. A background signal was measured and subtracted using a reference sample holder of the same dimensions filled with 0.6 g of deionized H_2_O.

A similar sample holder was used for QENS measurements of living housefly larvae [52]. The larvae of a total mass of 0.8 g were gently placed into a holder 30 mm wide, 70 mm deep, and 2 mm thick. The mass of the larvae sample matched the total sample mass in the previous measurements of planarians (0.2 g of flatworm specimens in 0.6 g of H_2_O) [51]. The sample holder was not sealed. A background signal was subtracted using an empty sample holder.

QENS measurements on living millipedes [53] used much larger individual specimens, weighing several grams each. Such large specimens had to be placed into cylindrical aluminum sample holders of 54 mm height and 29 mm inner diameter. A background signal was subtracted using an empty sample holder.

**Figure 3:**
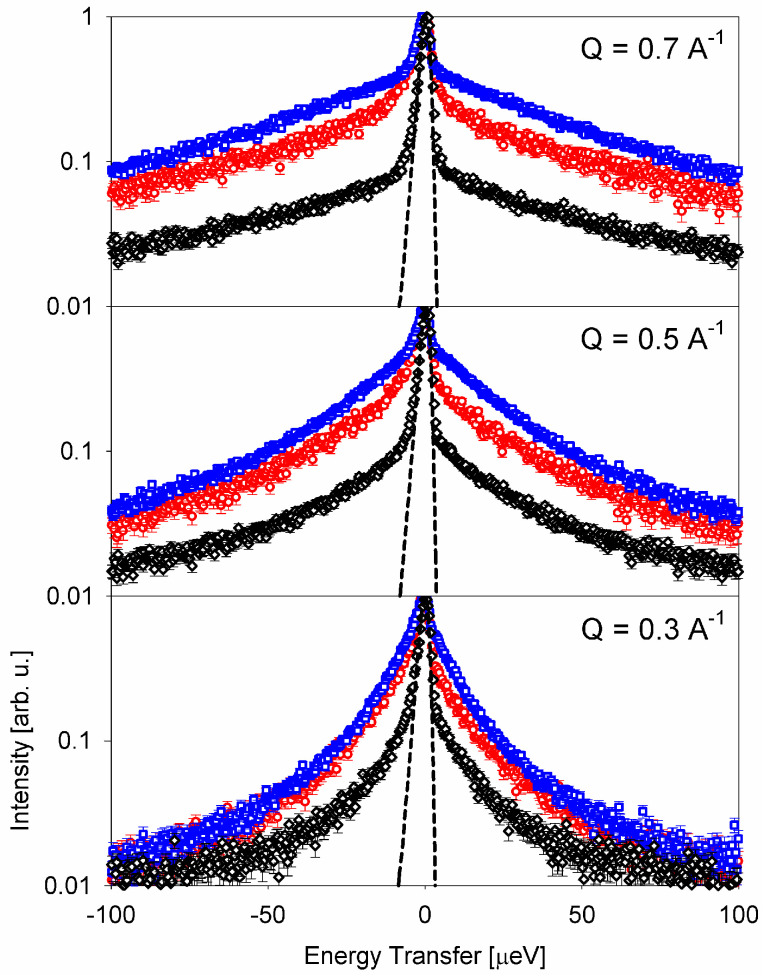
Scattering intensities I(Q,E) (background subtracted) measured at several Q values from planarian flatworms at 290.7 K [51] (red symbols), housefly larvae at 289.3 K [52] (blue symbols), and a millipede specimen at 290.0 K (black symbols). The resolution spectra are shown as the black dashed line. All spectra are normalized to unity. Error bars in all figures represent one standard deviation. Reprinted from [53].

Figure 3 shows a comparison of the scattering intensities (with the peak intensity normalized to unity) measured from different species at a neutron backscattering spectrometer, BASIS, at Oak Ridge National Laboratory’s Spallation Neutron Source [54]. In the default operation mode, BASIS has an energy resolution of ca. 3.5 µeV (full width at half maximum, FWHM) and an energy transfer range of ±100 µeV, corresponding to an accessible time range of ca. 6 to 400 ps. It is evident that, compared to the soft-bodied flatworms and larvae, the scattering signal from a millipede exhibits a relatively stronger narrow component centered at E = 0. As chitin, which is abundant in millipedes, is rigid and thus expected to give rise to a purely elastic signal on the energy/time scale of a QENS measurement, the more “elastic” appearance of the scattering signal from a millipede is rather intuitive. Indeed, while the QENS signal from flatworms and larvae could be well fitted using a superposition of two Lorentz functions (convolved with the resolution), the spectrum from a millipede required an additional elastic component for a satisfactory fit. The broader of the two Lorentz components measured in all the organisms exhibited a jump diffusion mechanism and was associated with “free” cytoplasmic water. On the other hand, the narrower of the two Lorentz components measured in the flatworms and larvae exhibited slow dynamics in the μeV range obeying a continuous (Fickian) diffusion law, with the QENS broadening proportional to DQ^2^, where D is the diffusion coefficient. A continuous diffusion mechanism would be unusual to observe for water, either free or bound, but is commonly seen in measurements of lateral diffusion in lipid membranes or in lipoproteins [55]. While unambiguous assignment of the narrow (slow) dynamic component measured from such complex systems as living organisms would be far from certain, it has been argued [51,52,53] that this motion is most likely associated with lipids. Importantly, the width of the narrow component was maintained much more tightly compared to the width of the broad component through the investigated range of physiological temperatures. That is, while the cytoplasmic water dynamics readily respond to changes in the environment temperature, the dynamics of the lipids remain tightly regulated. Both the global (center-of-mass) and the local (side chains) motions of proteins would be coupled to the temperature-sensitive dynamics of water. This notion, together with DQ^2^ dependence of the signal broadening, was instrumental in ascribing the slow dynamic component to lipids.

For the soft-bodied flatworms and larvae, the temperature dependence of the diffusion coefficient, D, obtained from the fits of the narrow component broadening with DQ^2^ law, could be displayed directly. On the other hand, the narrow Lorentz component measured from a millipede was also in the μeV range, but lacked a pronounced Q-dependence, likely due to the greater overall complexity of the organism, thus precluding straightforward quantification of the temperature dependence of the slow dynamics. Therefore, an “elastic” intensity scan was employed at a neutron backscattering spectrometer, HFBS, at NIST Center for Neutron Research [56], to probe the temperature dependence of the slow dynamics in the millipede specimen. These measurements utilized the same cylindrical sample holders as the measurements of a millipede at BASIS.

**Figure 4:**
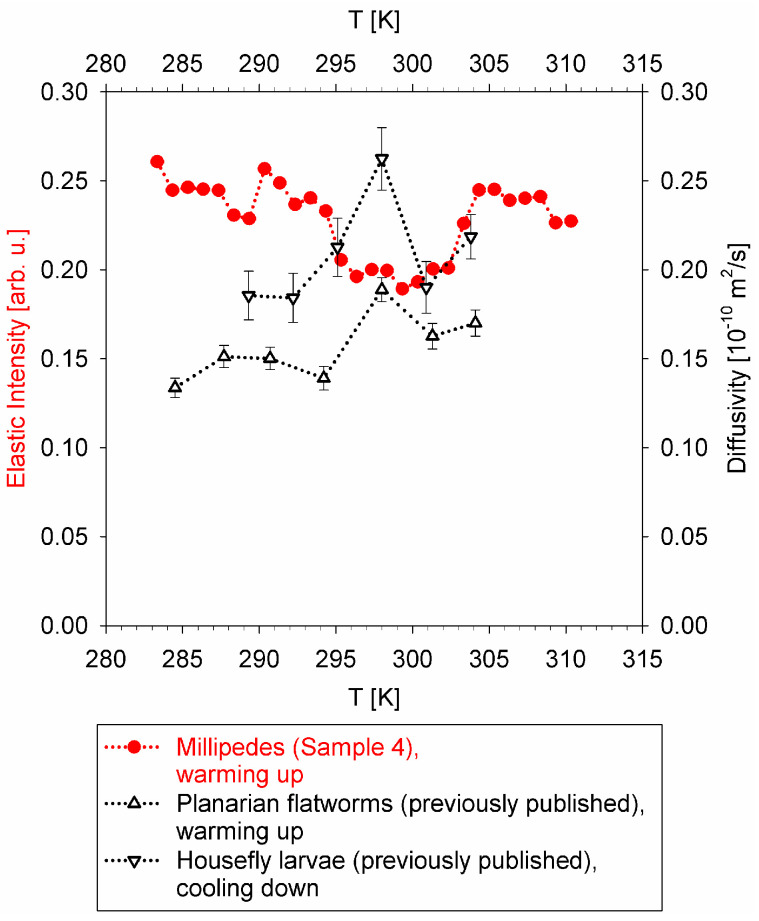
Red filled circles: temperature dependence of the elastic scattering intensity (values shown on the left axis) from millipede sample on warming up at 0.2 K/min. Error bars are within the symbols. Open up-triangles: microscopic diffusivity (values shown on the right axis) measured for planarian flatworms on warming up [51]. Open down-triangles: microscopic diffusivity measured for housefly larvae on cooling down [52]. Dotted lines are a guide for the eye. The data were summed up for 0.25 Å^−1^ < Q < 1.75 Å^−1^. Reprinted from [53].

Despite some scatter in the data presented in Figure 4, the non-monotonic temperature dependence of the diffusivities (black symbols) and “rigidity” (“elastic” scattering intensity, red symbols) is evident. The diffusivities measured in flatworms and larvae exhibit a maximum in the mid-temperature range, while the elastic scattering intensity shows a minimum in the same mid-temperature range. Remarkably similar temperature dependence of the datasets collected in different types of measurements at different spectrometers using different sample environment equipment in each experiment, and obtained either on warming up or cooling down, suggests that the non-monotonic temperature dependence of the slow dynamics in the studied organisms is highly unlikely to be an artifact of the measurements.

**Figure 5:**
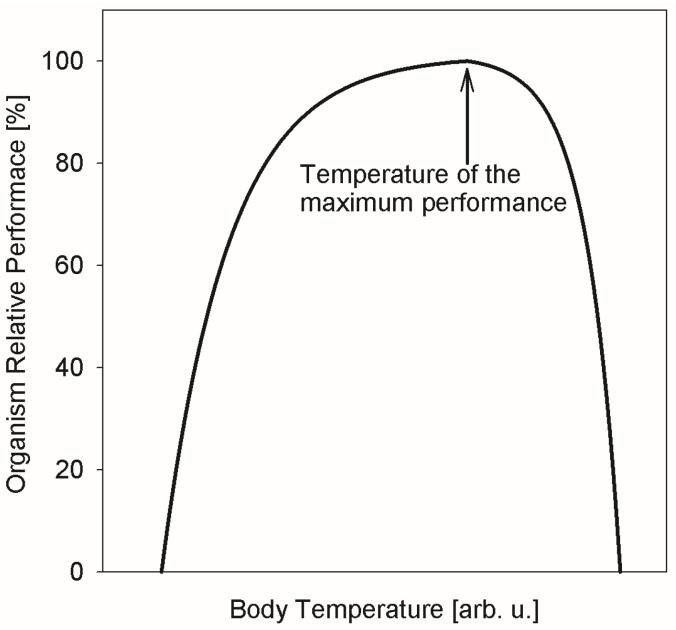
Schematic performance curve of an ectothermic organism as a function of temperature (as described in [57]. Reprinted from [53].

Thus, these results suggest the existence of a biological mechanism, possibly common between different classes (Insects and Myriapods) and even phyla (Arthropods and Platyhelminthes), that governs the slow nanoscopic dynamics in ectothermic organisms in response to the temperature of their environment. The boundaries of the physiologically acceptable temperature range are usually evident from the ectothermic animal’s “performance curve”, as presented schematically in Figure 5. The performance curve is an asymmetric function with respect to its maximum. The range between the critical thermal minimum and the maximum performance temperature exceeds the range between the maximum performance temperature and the critical thermal maximum [57]. The optimal temperature of the maximum performance does not coincide with the critical thermal maximum, demonstrating that an organism’s performance does not simply increase as a function of temperature according to the Boltzmann’s factor, exp(−E_a_/k_B_T), all the way up until the organism can become damaged by high temperature [58]. Instead, there is a decrease in the performance above the optimal temperature, which is due to the biological thermal stress factors [58] that can override the thermal activation-driven metabolic rate increase. It is possible that a mid-range temperature maximum in the diffusivity, or a minimum in the “rigidity”, as observed in the QENS experiments, is a manifestation of such thermal stress factors, which, upon exceeding the temperature optimal for an organism, put a break on any further increase in the nanoscopic dynamics despite the increased environment temperature. The exact physiological mechanisms responsible for slowing down the nanoscopic dynamics are difficult to pinpoint, but we note that, for example, cholesterol entering a phospholipid membrane makes it more rigid and suppresses the lipid diffusivity in the fluid phase of the membrane [59].

## 5. Perspectives on Incoherent Neutron Spectroscopy Studies of Complex Biological Systems in View of Advances in Spectrometer Design

The capability of neutron spectroscopy of measuring living organisms is remarkable among spectroscopic probes. Not only is the interaction of thermal or cold neutrons with the samples predominantly non-ionizing, thus enabling studies of relatively thick living specimens, but also the flux of neutrons incident on a sample is relatively low, limited by the neutron source. For instance, even the projected advanced neutron spectrometer, BWAVES, designed for simultaneous measurements of QENS and INS signals (hence, featuring a broadband, almost “white” incident neutron spectrum) is expected to have an incident flux of ca. 10^9^ neutrons per second per centimeter squared, with a 0.25 cm^2^ sample [60]. A canonical water sample, which is ca. 0.1 mm thick to ensure at least 90% neutron transmission and has a density of 1 g/cm^3^, would have ca. 10^20^ molecules. That is, in the course of an hour-long measurement, only a tiny fraction of molecules in the sample will interact with the incident neutron beam, characterized by 0.25 × 10^9^ neutrons per second = 9 × 10^11^ neutrons per hour flux. While biomacromolecules in a sample can be much larger in size than water molecules, most of them will not interact with a neutron during an hour-long measurement. This is in a dramatic contrast with high-flux photon- and electron-based probes, which, additionally, are highly ionizing due to their direct interaction with the electron shells. A commonly cited notion is that, for an individual neutron, the atomic scattering cross-sections are small, enabling the use of relatively thick samples, but it is also true that most molecules in a sample will not interact with neutrons due to the rather limited incident flux, making neutron scattering a gentle probe of living systems.

Compared to, e.g., nuclear magnetic resonance-based techniques, neutron scattering techniques exhibit a signal Q-dependence that can help decipher the geometry of the molecular motions but lack the site-specific sensitivity. Instead, QENS and INS measure the cross-section weighted dynamic characteristics of an entire sample, albeit on the atomic scale (defined by the scattering momentum transfer, Q), which can be linked to the sample’s thermodynamic parameters. For example, changes in the free energy upon protein–ligand binding are related to changes in the vibrational density of states [61], as commonly measured by INS, and conformational entropy changes upon protein unfolding are linked to the mean-squared atomic displacements, as measured by QENS [62]. In addition, phase transitions are evident from temperature scans of neutron “elastic” scattering intensity. In general terms, inelastic neutron scattering measures the imaginary part of the dynamic susceptibility of the entire sample, which is conceptually similar to thermodynamic response functions. Thus, with the advent of neutron spectrometers capable of measuring QENS and INS signals simultaneously [60], studies of the thermodynamics of living organisms may become possible.

One could question how much useful information can be retained when the scattering pattern from a biological system of enormous complexity is reduced to a scalar function of just two variables: the scattering energy and momentum transfer. To appreciate the usefulness of neutron spectroscopy measurements of living organisms, one should recall a routinely accepted but nevertheless truly remarkable fact that a single variable such as body temperature can be used to evaluate the health of an animal, an enormously complex multicellular organism. This is due to homeostasis, a regulatory mechanism which tightly maintains the stability of many physiological characteristics of an organism, out of equilibrium with the ever-changing environment. As mentioned above, neutron scattering measurements have revealed that microscopic diffusivity measured in living animals is surprisingly well defined and can be separated into that of intracellular cytoplasmic water and of the other cell constituents. Moreover, the dynamics of the latter are maintained much more tightly compared to the former through the investigated range of physiological temperatures. Thus, besides widely recognized tightly maintained homeostatic parameters such as temperature, pH, osmolality, etc., microscopic diffusivity in the cell constituents may also be maintained in a living organism, even when the intracellular water readily responds to the temperature changes in the environment. As microscopic diffusion of cell constituents is vital for molecular transport and metabolic activity at the cellular and intra-cellular level, its measurement demonstrates the role the neutron spectroscopy measurements of living organism could uniquely play.

In view of the proven success of studies of preserved tissues, another promising direction of incoherent neutron spectroscopy might involve studies of living tissues in higher organisms. However, the practical limitations to this approach might arise due to the sample thickness. Even though multiple scattering effects in relatively thick biological samples have proven manageable, with increasing sample thickness, the beam attenuation will eventually render scattering measurements in transmission geometry outright impossible.

To summarize, neutron scattering can be applied as a gentle probe of living systems, and recent studies have demonstrated that valuable information may be obtained from incoherent neutron spectroscopy measurements of biological systems of the highest complexity, such as living multicellular organisms. The frontier of biological complexity amenable to incoherent neutron spectroscopy studies is presently expanding from the cellular to multicellular (tissues and organisms) level. With the aid of upcoming neutron spectrometers (e.g., [60]), further advances in incoherent neutron spectroscopy of complex biosystems can be expected.

## Data Availability

This review discusses the data previously published in the literature.
